# Modeling Opposite Effects of an Additive on Liquid–Liquid Phase Separation and Crystal Solubility of Protein Solutions

**DOI:** 10.3390/molecules31111894

**Published:** 2026-06-01

**Authors:** Onofrio Annunziata, Shamberia Thomas

**Affiliations:** Department of Chemistry and Biochemistry, Texas Christian University, Fort Worth, TX 76109, USA

**Keywords:** lysozyme, HEPES, NaCl, metastability, anisotropy, Barker–Henderson

## Abstract

In protein solutions, an additive that increases protein–protein attractive interactions is expected to decrease protein crystal solubility and raise the temperature at which liquid–liquid phase separation (LLPS) occurs. In contrast, addition of 0.10 M 4-(2-hydroxyethyl)-1-piperazineethanesulfonate (HEPES) to lysozyme–NaCl aqueous solutions at constant pH (7.4) and ionic strength (0.20 M) decreases solubility but lowers the LLPS temperature. This leads to the broadening of the LLPS metastability gap in the phase diagram and an enhancement of protein crystallization yield from LLPS. We theoretically examine the effect of HEPES on both solubility and LLPS boundaries using a colloid model. Under the hypothesis that HEPES stabilizes protein–protein contacts in the crystal lattice by physical cross-linking, we apply cell theory to describe the thermodynamic behavior of the crystalline phase and use solubility data to show that HEPES increases protein–protein attraction energy by 2.7%. Since an increase in attraction incorrectly predicts a rise in the LLPS temperature, we consider that HEPES also enhances the anisotropic character of protein–protein interactions. To describe the thermodynamic behavior of the solution phase, we start from Barker–Henderson second-order perturbation theory on the hard-sphere reference fluid with square-well potential and local-compressibility approximation. We modify this model so that it can reproduce the correct mathematical expression of the second virial coefficient. This also leads to better agreement with Monte Carlo simulations. We then approximately incorporate anisotropy by assuming that the square-well attraction energy is a temperature-dependent average over all the surface of a particle with a given fractional coverage of attractive spots. The attraction energy of the attractive spots is set to be the same as that of the protein–protein contacts in the crystal. Only fractional coverage (anisotropy) was varied to successfully fit the effect of HEPES on the LLPS boundary.

## 1. Introduction

Understanding and controlling condensation of globular proteins in aqueous media is fundamental for comprehending cell compartmentalization [1,2], protein-aggregation diseases [3], developing stable pharmaceutical formulations [4,5], preparing protein-based materials [6], and generating protein crystals [7,8] with applications in structural biology and separation science.

An interesting phenomenon of protein aqueous mixtures is the reversible formation of metastable protein-rich liquid microdroplets through liquid–liquid phase separation (LLPS), typically induced by lowering temperature [9,10,11]. Since LLPS is a metastable phase transition, the protein-rich phase can act as an intermediate for the formation of other protein condensed phases such as crystals [10,12] and aggregates [3,13,14]. This phenomenon was initially considered for only a few protein cases: eye-lens crystallins [9,15,16] and lysozyme [10,17]. However, it has recently attracted considerably more attention as it is believed to drive the formation of membraneless organelles in the cytosol and can promote formation of pathological protein aggregates [1,2,3,18]. It has also been investigated in the context of protein pharmaceutical formulations, where the LLPS is described to negatively impact the stability and efficacy of monoclonal antibodies [4,5]. Finally, LLPS is also known to enhance the rate of protein crystallization [12,19,20,21], which is beneficial not only for the characterization of protein 3D structure [22] but also for protein purification in downstream processing [23].

From a molecular point of view, all protein condensation processes are driven by solvent-mediated protein–protein attractive interactions [24]. In principle, it is extremely difficult to describe protein–protein interactions as they ultimately depend on the surface composition and spatial orientation of amino-acid groups. These are responsible for the formation of a highly heterogeneous distribution of charged chemical groups, hydrophilic moieties and hydrophobic patches. Moreover, additives, which are invariably present in protein solutions, can significantly alter protein–protein interactions [25]. These cosolvents modify these interactions through mechanisms such as electrostatic screening [26], preferential hydration or binding [27,28], and molecular crowding [11,29,30]. Nevertheless, some key features associated with the collective behavior of globular proteins can be theoretically described by employing a simple one-component colloid model using hard spheres as a reference system [31,32,33]. For example, LLPS metastability with respect to protein crystallization is successfully explained by assuming that the range of protein–protein interactions is relatively short compared to particle diameter [31].

**Figure 1:**
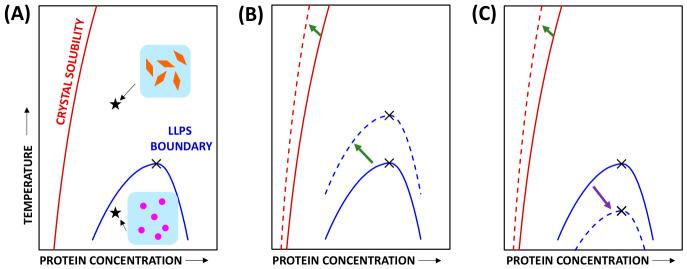
(**A**) Schematic temperature–concentration phase diagram showing crystal solubility (red) and LLPS boundary (blue; critical point, ×). The star symbols indicate two representative states with the same protein concentration. Both states are supersaturated with respect to protein crystallization and are expected to generate crystals (diamonds). The state at lower temperature is below the LLPS boundary and is associated with the formation of protein-rich spherical droplets (circles). (**B**) Schematic temperature–concentration phase diagram showing the effect of an additive that shifts the two phase boundaries toward higher temperatures (e.g., NaCl for lysozyme). (**C**) Schematic temperature–concentration phase diagram showing the effect of an additive that shifts the two phase boundaries in opposite directions thereby increasing metastability gap (e.g., HEPES for lysozyme).

From a thermodynamic point of view, the phase behavior of a protein aqueous system is typically described by employing a temperature–composition phase diagram. As schematized in Figure 1A, a typical phase diagram shows a protein-crystal solubility curve with the LLPS boundary positioned in the crystal supersaturated domain, at relatively high protein concentrations. Note that the LLPS boundary can be experimentally characterized because protein crystallization kinetics is usually slow [4,9,31,34].

Additives such as salts, buffer components, and polyethylene glycols are typically present in protein aqueous solutions. They are used to stabilize proteins against unfolding, favoring protein crystallization and mimic physiological conditions. The thermodynamic effect of additive concentration has often been described using the theory of preferential interaction [35]. Accordingly, an additive that is preferentially excluded from the vicinity of a protein surface (preferential hydration) reduces protein solubility (e.g., NaCl for lysozyme, sulfates). In contrast, an additive that accumulates near the protein surface (preferential binding) increases protein solubility (e.g., thiocyanates, urea) [36,37]. A one-component colloid model may still be used to describe the phase behavior of these multicomponent protein solutions provided that the additive has a low molecular weight and is therefore considered as an integral part of the solvent. The effect of additive concentration is then implicitly taken into account by considering a corresponding change in the solvent-mediated protein–protein interactions [33]. For example, attraction energy should increase with additive concentration in the presence of preferential hydration [38]. As illustrated in Figure 1B, this salting-out effect leads to a shift of both crystal solubility and the LLPS boundary towards higher temperatures, with crystal solubility shifting toward lower protein concentrations. This behavior has been experimentally demonstrated for lysozyme in the presence of NaCl and other additives [10,39,40]. Interestingly, shifts in the LLPS boundary appear to be stronger than those of crystal solubility. This results in an increase in the metastability gap between the LLPS boundary and solubility curve [40].

In our previous studies, we reported that addition of 4-(2-hydroxyethyl)-1-piperazineethanesulfonate (HEPES) to lysozyme–NaCl solutions, while maintaining the same pH and ionic strength, leads to an even more complex impact on the phase diagram [41,42]. As qualitatively schematized in Figure 1C, HEPES moves the solubility curve toward higher temperatures (as in Figure 1B) while moving the LLPS boundary in the opposite direction. This leads to a significant increase in the metastability gap between the two phase boundaries. Remarkably, addition of HEPES was also found to significantly increase the yield of protein crystallization, especially in the presence of LLPS [21]. Clearly, understanding the multifaceted effect of this type of additive on the phase behavior of protein solutions, especially in relation to the metastability gap, is important for controlling protein crystallization and other aggregation processes.

To explain the effect of HEPES on the lysozyme phase diagram, partitioning experiments were also carried out. It was found that HEPES accumulates in the protein-rich liquid phase, showing that HEPES preferentially binds to lysozyme [41]. Furthermore, dynamic light-scattering experiments showed that HEPES weakens protein–protein attraction energy [41]. While these results are consistent with HEPES suppressing lysozyme LLPS, they cannot explain the salting-out effect of HEPES on lysozyme solubility.

The lysozyme 3D structure obtained from crystals grown in HEPES buffer also shows that its organic molecules act as a ligand. Indeed, it is found in the lysozyme catalytic site, with the hydroxyethyl pointing inward [43]. On the other hand, the sulfonate group is found to be close to a cationic arginine group of a neighboring protein. It is therefore reasonable to assume that the electrostatic attraction between these two ionic groups may strengthen one of the crystal contacts [42]. In other words, the effect of HEPES on lysozyme solubility may be qualitatively explained by considering that this organic molecule acts as a physical cross-linker, enhancing attractive interactions between neighboring protein units in the crystal lattice. This thermodynamically stabilizes the crystalline phase. Our hypothesis aligns with the general belief that multifunctional organic molecules are beneficial in protein crystallography [44].

In this report, we investigate the ability of a colloid model to quantitatively describe the observed effects of HEPES on lysozyme phase behavior. We specifically model the effect of this additive by assuming that it produces an increase in protein–protein attractive interactions together with an increase in their anisotropic character. We further assume that HEPES does not alter protein conformation.

## 2. Results and Discussion

To describe the effect of HEPES on the phase behavior of lysozyme–NaCl–water mixtures, two specific systems are examined. The first system consists of HEPES (0.10 M) and NaCl (0.15 M) and is denoted as the HEPES system. The second system, which is a reference system (denoted as REF) consists mainly of NaCl (0.183 M; Tris buffer, 0.020 M) and share the same pH (7.4) and ionic strength (0.20 M) [41,42]. In this way, replacement of NaCl with HEPES is carried out under conditions in which long-range electrostatic repulsive interactions between proteins remain the same. Note that the presence of Tris buffer is REF, which was employed to improve buffer capacity. Although its contribution to the total ionic strength of 0.20 was taken into account, its concentration is fairly low and Tris-specific effects are not expected to be significant.

In the following subsections, we first review the thermodynamic framework employed for modeling the phase boundaries of protein solutions (Section 2.1). We next examine the effect of HEPES on lysozyme crystal solubility (Section 2.2), and then characterize the corresponding effect on the LLPS boundary (Section 2.3 and Section 2.4).

### 2.1. Thermodynamic Framework

An aqueous solution of protein is thermodynamically described as a one-component compressible fluid made of colloidal particles, provided that the aqueous fluid can be assumed as incompressible and all low-molecular-weight cosolutes such as salts and buffer components are implicitly considered as a part of the background solvent. These additives are modulators of solvent-mediated protein–protein interactions. Correspondingly, the protein osmotic pressure, Π, becomes a “gas pressure” and LLPS is equivalent to a “gas–liquid” phase transition. Furthermore, protein chemical potential, μ, must describe particle insertion accompanied with isochoric removal of solvent [24]. It is therefore appropriate to start from the Helmholtz free energy, A, of the colloid fluid. It is also practically convenient to introduce the corresponding reduced unitless quantity, $a\equiv \beta (V_{P}/V)A$, where $\beta \equiv 1/k_{B}T$, $k_{B}$ is the Boltzmann constant, T the absolute temperature, $V_{P}$ the protein volume, and V is the volume of the fluid phase. We set $k_{B}=1$ so that $\beta^{-1}$ is the same as temperature, and energy parameters adopt temperature units. The composition of the fluid phase is described by the volume fraction, $\phi =NV_{P}/V$, where N is the number of colloidal particles. An experimental volume fraction of lysozyme solution is readily calculated by multiplying lysozyme mass concentration by the known [45] specific volume of this protein (0.713 cm^3^·g^−1^).

The protein phase diagram [31] shows protein volume fraction (ϕ) and temperature ($\beta^{-1}$) on the *x*- and *y*-axis, respectively. The LLPS boundary is described by the protein volume fractions, $\phi^{\left(I\right)}(\beta )$ and $\phi^{\left(\mathrm{II}\right)}(\beta )$, of the two coexisting of protein-diluted (“gas”, I) and -concentrated (“liquid”, II) fluid phases. These two volume fractions become equal at the critical point, which is described by the critical coordinates, $\left(\phi_{c},\beta_{c}\right)$. To determine $\phi^{\left(I\right)}(\beta )$ and $\phi^{\left(\mathrm{II}\right)}(\beta )$, the expressions of chemical potential and osmotic pressure are extracted from the Helmholtz free energy using:

(1a) $$\hat{\mu }={\left(\frac{\partial a}{\partial \phi }\right)}_{\beta }$$

(1b) $$\hat{\pi }=\phi \;\hat{\mu }-a$$

where $\hat{\mu }\equiv \beta \mu$ and $\hat{\pi }\equiv \beta \;\Pi V_{P}$. At any sufficiently high value of β, the protein volume fractions, $\phi^{\left(I\right)}$ and $\phi^{\left(\mathrm{II}\right)}$, of the two coexisting phases must satisfy the chemical equilibrium conditions:

(2a) $$\hat{\mu }(\phi^{\left(I\right)},\beta )\;\;=\;\;\hat{\mu }(\phi^{\left(\mathrm{II}\right)},\beta )\;$$

(2b) $$\hat{\pi }(\phi^{\left(I\right)},\beta )\;\;=\;\;\hat{\pi }(\phi^{\left(\mathrm{II}\right)},\beta )\;$$

Critical coordinates, $\left(\phi_{c},\beta_{c}\right)$, satisfy the conditions: ${\left(\partial \hat{\mu }/\partial \phi \right)}_{\beta_{c}}\;=\;\;{\left(\partial^{2}\hat{\mu }/\partial \phi^{2}\right)}_{\beta_{c}}=0$.

The hard-sphere model is employed as a reference model. The reduced Helmholtz free energy, a, is then given by:

(3) $$a=a_{\mathrm{HS}}+a_{R}$$

where $a_{\mathrm{HS}}$ is the hard-sphere term, describing both particle translational motion (ideal contribution) and excluded-volume interactions (steric repulsion). It is given by:

(4) $$a_{\mathrm{HS}}(\phi )=\phi \;{\hat{\mu }}_{0}+\phi (\ln\phi -1)+\frac{4-3\phi }{{\left(1-\phi \right)}^{2}}\phi^{2}$$

where ${\hat{\mu }}_{0}$ is the uninfluential standard chemical potential, the second term represents the ideal-gas contribution and the third term describes steric repulsion according to the Carnahan–Starling equation of state [46]. It follows from Equation (1a,b) that the hard-sphere chemical potential and pressure are given by ${\hat{\mu }}_{\mathrm{HS}}=\;{\hat{\mu }}_{0}+\ln\phi +(8-9\phi +3\phi^{2})\phi /{\left(1-\phi \right)}^{3}$ and ${\hat{\pi }}_{\mathrm{HS}}=\phi (1+\phi +\phi^{2}-\phi^{3})/{\left(1-\phi \right)}^{3}$, respectively. In Equation (3), $a_{R}$ is a residual term describing the deviation from the reference model. It characterizes protein–protein attraction energy and vanishes in the limit of high temperatures (β→0) [47]. This residual term is further discussed in Section 2.3.

The crystalline ordered phase, which has a very high protein volume fraction (typically more than 50%), is assumed to be incompressible [31,48]. In this case, the protein chemical potential in the solid crystalline phase, $\mu_{S}(\beta )$, coincides with the Helmholtz free energy of one particle. The crystal solubility boundary is described by the protein volume fraction of the coexisting fluid phase, $\phi_{S}(\beta )$. At any given β, this is determined by numerically solving the chemical-equilibrium condition:

(5) $$\hat{\mu }(\phi_{S},\beta )\;={\hat{\mu }}_{S}(\beta )$$

where ${\hat{\mu }}_{S}\equiv \beta \mu_{S}$. A simple expression for ${\hat{\mu }}_{S}$ was obtained from cell theory as discussed in the next section.

The second virial coefficient, B in ${\hat{\pi }}_{\mathrm{HS}}/\phi =1+B\;\phi +\ldots$, is a fundamental thermodynamic parameter that quantifies the strength of protein–protein net interactions. It is therefore directly relevant to both LLPS and crystal solubility [49]. A negative value of B indicates that net interactions between proteins are attractive and is necessary for LLPS and protein crystallization to occur. Thus, a thermodynamic model describing LLPS and crystallization boundaries must invariably examine the corresponding behavior of the second virial coefficient.

Finally, although the one-component thermodynamic description of a colloid system does not take into account protein-additive specific interactions explicitly, it does not mean that it neglects their contribution. Indeed, it is possible to thermodynamically link $\partial \hat{\mu }/\partial \phi$ and B to the preferential-interaction coefficient [38].

### 2.2. Crystal Solubility and Thermodynamics of Protein Crystal

To theoretically describe the solubility boundary using Equation (5), we need the mathematical expressions for the protein chemical potential in the crystal and fluid phases. According to cell theory [31,48], ${\hat{\mu }}_{S}$ may be written as:

(6) $${\hat{\mu }}_{S}={\hat{\mu }}_{0}-n_{S}\frac{\beta \varepsilon_{S}}{2}-\ln\Omega_{S}$$

where ${\hat{\mu }}_{0}$ is the same as in Equation (4). The second term represents the internal energy of the crystal, where $n_{S}$ is the number of interaction “sites” or “contacts” between a central protein and its neighboring surrounding proteins. The factor “2” considers that a protein–protein interaction involves two proteins; i.e., the range of interactions is sufficiently short that one interaction cannot extend to more than two proteins. In Equation (6), $\varepsilon_{S}$ is the average attraction energy of a contact. The definition of the number of contacts is somewhat arbitrary. Indeed, an individual contact could be defined as involving a single functional group or as a relatively large region on the protein surface involving multiple functional groups on the protein surface. Thus, the value of $\varepsilon_{S}$ depends on the choice of $n_{S}$ and only the product, $n_{S}\varepsilon_{S}$, can be unambiguously defined. The last term in Equation (6) is an entropic term that takes into account the residual translational and orientational motion of a protein inside the crystal lattice, with $\Omega_{S}$ being the phase volume [48] (as a multiple of particle volume, $V_{P}$) accessible to both the particle center of mass and particle rotation. The extracted value of $\Omega_{S}$ is discussed further at the end of this subsection.

The highest experimental [42] $\phi_{S}$ are 0.014 and 0.027 for the HEPES and REF system, respectively. We assume that these values are sufficiently low that the protein chemical potential of the fluid phase is approximated by its ideal-dilute approximation, $\hat{\mu }\approx {\hat{\mu }}_{0}+\ln\phi$, neglecting the contribution of protein–protein interactions in the fluid phase. We later validate this assumption by verifying that inclusion of steric repulsion and protein–protein attraction energy terms do not significantly alter the solubility boundary. Insertion of Equation (6) into Equation (5) yields the following van’t Hoff equation:

(7) $$\ln\phi_{S}=-n_{S}\frac{\beta \;\varepsilon_{S}}{2}-\ln\Omega_{S}$$

**Figure 2:**
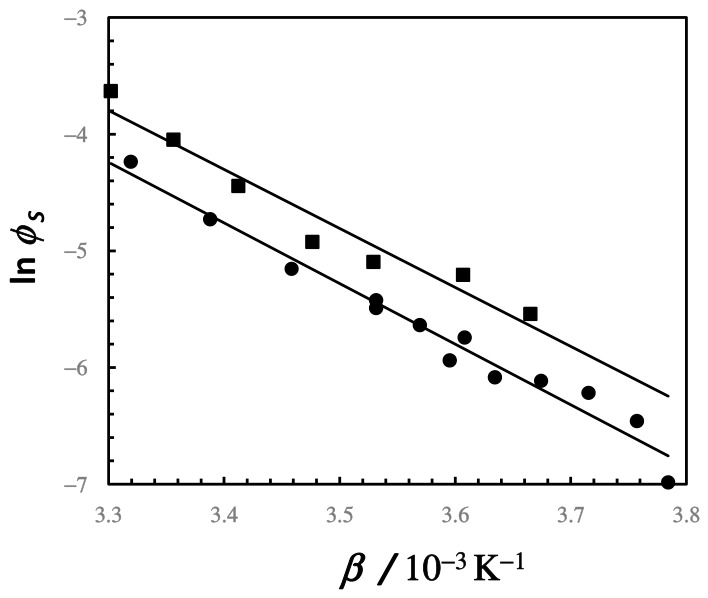
Van’t Hoff plot of crystal solubility, *ϕ_S_*, as a function of inverse temperature, *β*, for lysozyme in HEPES (circles) and REF (squares) systems. Solid lines are linear fits through the data based on Equation (7).

Van’t Hoff plots for lysozyme solubility in the HEPES and REF systems are shown in Figure 2. Here, we can see that solubility values in the HEPES system are appreciably lower than those in the REF system. Application of the method of least squares yields: $n_{S}\varepsilon_{S}=$ (10.4 ± 0.5)10^3^ K (86.6 kJ·mol^−1^) and $\ln\Omega_{S}=$ −(12.9 ± 1.0) for the HEPES system and $n_{S}\varepsilon_{S}=$ (10.1 ± 1.0)10^3^ K (84.0 kJ·mol^−1^) and $\ln\Omega_{S}=$ −(12.9 ± 1.8) for the REF system. These values are essentially the same within the experimental errors due to correlation between the slope and intercept parameters. To further examine these solubility data, we assume that addition of HEPES has a negligible effect on crystal entropy. This assumption is motivated by the hypothesis that HEPES energetically stabilizes region contacts through physical cross-linking while not appreciably altering crystal lattice, consistent with available crystal structures [43,50,51]. Accordingly, we define $n_{S}$ as a region contact and assume it is the same for both systems. We then set $\ln\Omega_{S}=$ −12.9 so that we can attribute solubility variations entirely to $\varepsilon_{S}$. We can now determine appreciably different values of $n_{S}\varepsilon_{S}$. These are (10.39 ± 0.02) × 10^3^ K and (10.12 ± 0.03) × 10^3^ K for the HEPES and REF systems, respectively. The corresponding linear fits are also shown in Figure 2. These share the same intercept at β = 0.

**Figure 3:**
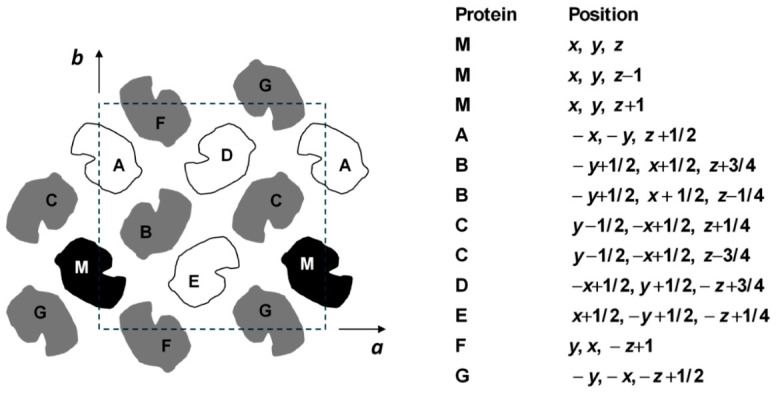
Simplified representation of lysozyme proteins on the (0,0,1) crystallographic plane as described in the literature [51,52]. Dashed box represents the unit cell along the *a*- and *b*- crystallographic axis. Each type of protein is labeled using a letter: reference protein (M; black), proteins interacting with M (B, C, G, and F; gray) and proteins non-interacting with M (A, D, and E; white). Positions (and orientations) of proteins relative to M (*x*,*y*,*z*) are also listed.

A reasonable value of the number of contacts, $n_{S}$, is needed to determine $\varepsilon_{S}$. It has been reported [51,52] that a lysozyme molecule interacts with eight neighboring lysozyme molecules in a tetragonal crystal structure. This implies that there are eight region contacts on each protein engaging in multiple interatomic bonds with the surrounding proteins. However, it has been established [52] that the interactions with the two proteins immediately above and below the reference protein (along the crystallographic *c*-axis) are relatively weak and can be therefore neglected. The proteins involved in the interactions with a reference protein (M) are represented on the (0, 0, 1) crystallographic plane shown in Figure 3. Here, we can appreciate that there are two neighboring proteins, F (at −z+1) and G (at −z+1/2), related to M by twofold screw symmetry. There are also two B (at z+3/4 and z−1/4) and two C (at z+1/4 and z−3/4) proteins with different positions along the *c*-axis interacting with M. These other four proteins are related by fourfold screw symmetry to M [51,52]. The catalytic site (cleft) of protein M, which can be occupied by HEPES, is near the protein B at z−1/4. It is therefore reasonable to attribute the increase in attraction energy caused by HEPES to this specific contact region.

In summary, there are six main region contacts on the protein that can be identified as binding sites. We therefore set $n_{S}=$ 6 and determine that the average attraction energy of an interacting region is $\varepsilon_{S}=$ 1732 K and 1687 K for the HEPES and REF systems, respectively. In summary, our data analysis shows that HEPES causes an increase of 2.7% in the value of $\varepsilon_{S}$ inside the crystal.

We conclude this subsection by examining the extracted $\ln\Omega_{S}$ describing crystallization entropy. Although its accurate interpretation should factor in protein shape and changes in solvent entropy, it is important to examine whether the value of phase volume, $\Omega_{S}=2.5\times 10^{-6}$, yields physically acceptable geometric parameters. We specifically assume that $\Omega_{S}$ is the product of a translational factor, $\Omega_{r}$, and an orientational factor, $\Omega_{\theta }$. The translational factor is given by $\Omega_{r}=V_{r}/V_{P}$, where $V_{r}$ is the center-of-mass excursion volume. If proteins are assumed to be spherical particles, we can use unit cell volume of tetragonal lysozyme (238 nm^3^), number of proteins inside unit cell (8) [51], and protein volume (16.9 nm^3^) to determine that the protein volume fraction in the crystal is 0.57. For spherical particles, translational motion is lost when the volume fraction reaches the classical close-packing value of 0.7405. Since an increase of 1.091 in particle diameter is needed to increase the experimental volume fraction to the close-packing value, the diameter of the excursion sphere is estimate to be 2×0.091 σ, where σ is the diameter of the protein. This implies that $\Omega_{r}\approx 6.0\times 10^{-3}$ from which we calculate: $\Omega_{\theta }\approx 4.1\times 10^{-4}$. To estimate the angular degree of freedom, we may set: $\Omega_{\theta }\approx \theta^{\;3}/(8\pi^{2})=4.1\times 10^{-4}$, where $8\pi^{2}$ represents the maximum angular phase corresponding to a freely rotating particle, and θ is the geometric mean of the three Euler angles accessible through trough particle rotation in the crystal [48,53]. We then extract θ≈ 18°, which is a physically acceptable [48] angular degree of freedom.

### 2.3. Thermodynamic Model for the Fluid Phase

In order to describe the thermodynamic behavior of protein solutions, an expression for $a_{R}$ in Equation (3) is needed. To enable LLPS, this expression must incorporate protein–protein attraction energy. According to our solubility results, HEPES is responsible for an increase in protein–protein attraction energy. This increase, however, should also cause a corresponding increase in the LLPS temperature, in contrast with experimental results showing that HEPES causes a ≈5% decrease [41,42] in the LLPS temperature.

To further examine the effect of HEPES on protein–protein interactions, we consider previous measurements of lysozyme diffusion coefficient [41] as a function of protein concentration for the HEPES and REF systems. Assuming that the hydrodynamic factor [54] is the same for both systems, these diffusion data show that HEPES causes an increase of 1.2±0.4 in the second virial coefficient at 298 K [32]. This confirms that HEPES decreases protein–protein attraction energy in the fluid phase. It is in apparent conflict with HEPES being able to increase protein–protein attraction energy in the crystalline phase.

We previously mentioned that HEPES can stabilize lysozyme crystals by physical cross-linking interactions. This type of interaction can also occur in the fluid phase. However, their highly directional character makes them entropically unfavored, i.e., cross-linking can occur only if the relative orientation between two proteins is appropriate. In the crystalline phase, this entropy cost is marginal because protein orientation is essentially fixed by the lattice structure. In contrast, protein–protein attraction energy in the fluid phase can be weaker on average due to free rotation. In other words, it is the anisotropic character of protein–protein interactions that may explain the opposite effects of HEPES on lysozyme solubility and LLPS.

To theoretically derive LLPS boundaries, we need to discuss $a_{R}$ in Equation (3). There are several Monte Carlo studies accurately describing the Helmholtz free energy of model fluid systems appropriate for protein solutions [24,53,55,56,57,58,59]. Nonetheless, it remains practically convenient to use thermodynamic-perturbation theories [33,47,48,60,61,62] that provide an approximate analytical expression of the Helmholtz free energy. In our case, we consider a model that depends on just three parameters, describing attraction energy, range of interactions, and degree of anisotropy. These are linked to the three main topological features of a dome-shaped LLPS boundary: the two critical point coordinates, $\left(\phi_{c},\beta_{c}^{-1}\right)$, and the boundary width.

In this work, we consider the Barker–Henderson perturbation theory of square-well fluids (BHPT) [47,62] to describe the effect of HEPES on lysozyme phase behavior. Note that BHPT treats particle–particle interactions as isotropic. However, anisotropy may be incorporated in BHPT by introducing a temperature-dependent energy parameter that depends on the fraction of protein surface engaging in protein–protein interactions [53]. Our revised BHPT model is further discussed below. It is important to note that Wertheim perturbation theory of associating spheres (WPT) [33,48,60,63] has also been applied to examine the phase behavior of protein solutions. WPT explicitly describes anisotropic interactions by considering a specified number of sites that can link two spherical particles. However, we chose to use BHPT instead of WPT as there is a noticeable difference in $\phi_{c}$ between WPT and computer simulation data [24,59] on spherical particles with the same number of sites. Moreover, it also yields a relatively large difference in the second virial coefficient between the HEPES and REF systems. Nonetheless, for completeness, application of WPT with three parameters is discussed together with the corresponding second virial coefficient in the Supplementary Materials.

The residual Helmholtz free energy for the original BHPT model is written as:

(8) $$a_{R}(\beta ,\phi )=a_{\mathrm{mf}}+a_{R2}$$

where $a_{\mathrm{mf}}(\beta ,\phi )$ rigorously represents a mean-field first-order correction to the hard-sphere reference model. In the case of isotropic square-well potential, we have [47]:

(9) $$a_{\mathrm{mf}}=-\frac{1}{2}\;\nu_{\mathrm{HS}}\;\phi \;\beta \;\varepsilon_{\mathrm{SW}}$$

where $\varepsilon_{\mathrm{SW}}$ is the magnitude of the square-well attraction energy. Attraction occurs when the distance between the centers of two particles, r, is such that σ≤r≤λσ, while no interactions occur if r>λσ, where σ is the particle diameter and λ specifies the range of attractive interactions. In Equation (9), $\;\nu_{\mathrm{HS}}$ is the average number of contacts that each particle makes within the range 1≤x≤λ, evaluated using the pair distribution function of the reference hard-sphere fluid, $g_{\mathrm{HS}}(x,\phi )$ with x≡r/σ. We specifically write:

(10) $$\nu_{\mathrm{HS}}(\phi ,\lambda )=24\phi \int_{1}^{\lambda }g_{\mathrm{HS}}(x,\phi )x^{2}dx=8\;(\lambda^{3}-1)\phi \cdot g_{\mathrm{HS}}(1,\phi^{\prime })$$

where $8\;(\lambda^{3}-1)\phi$ represents $\nu_{\mathrm{HS}}$ in the limit of ϕ→0 and $g_{\mathrm{HS}}(1,\phi^{\prime })=(1-\phi^{\prime }/2)/{\left(1-\phi^{\prime }\right)}^{3}$ is the Carnahan–Staling contact value of $g_{\mathrm{HS}}(x,\phi^{\prime })$ evaluated at the particle volume fraction, $\phi^{\prime }(\phi ,\lambda )$. The following Padé approximant of $\phi^{\prime }(\phi ,\lambda )$ is available, [62,64]:

(11) $$\phi^{\prime }=\frac{c_{1}+c_{2}\;\phi }{{\left(1+c_{3}\;\phi \right)}^{3}}\phi$$

where $c_{i}=\sum_{j=1}^{4}s_{ij}\lambda^{-j}$ with i=1,2,3 and the 3×4 matrix of $s_{ij}$ coefficients is given by [−3.1649, 13.3501, −14.8057, 5.7029; 43.0042, −191.6623, 273.8968, −128.9334; 65.0419, −266.4627, 361.0431, −162.6996].

The second contribution in Equation (8), $a_{R2}$, describes the deviation of $a_{R}$ from the mean-field first-order contribution and can be evaluated only approximately. The most successful approximation is the local compressibility approximation [47,62], where $a_{R2}$ is the second-order perturbation term of the Helmholtz free energy. It was deduced by examining the radial profile of fluctuations of particle density around a central particle [47]. At a given radial distance, these fluctuations are approximately described by the isothermal compressibility of the hard-sphere reference model. It can be then shown that [47,62]:

(12) $$a_{R2}=-\frac{1}{2}\phi^{2}\frac{d\nu_{\mathrm{HS}}}{d{\hat{\pi }}_{\mathrm{HS}}}\frac{\beta^{2}\;\varepsilon_{\mathrm{SW}}^{2}}{2}$$

where $d\nu_{\mathrm{HS}}/d{\hat{\pi }}_{\mathrm{HS}}=(d\nu_{\mathrm{HS}}/d\phi )(d\phi /d{\hat{\pi }}_{\mathrm{HS}})$ and $\beta^{2}\;\varepsilon_{\mathrm{SW}}^{2}/2$ represents the second-order term in the series expansion of the Mayer function, $e^{\beta \varepsilon_{\mathrm{SW}}}-1$. We can numerically calculate $d\nu_{\mathrm{HS}}/d\phi$ from Equation (10) while using the Carnahan–Starling equation of state [46,62] to determine that $d\phi /d{\hat{\pi }}_{\mathrm{HS}}={\left(1-\phi \right)}^{4}/(1+4\phi +4\phi^{2}-4\phi^{3}+\phi^{4})$. It is important to note that the BHPT model is applicable for moderately short interaction ranges, λ≈1.2 and higher [62]. This is appropriate for protein solutions with $\phi_{c}\approx 0.22$ and less [24]. It should not be used when particle–particle interactions are very short (λ<1.2).

The range of interaction, λ, is the only parameter that affects the value of the critical volume fraction, $\phi_{c}$. In our application of the BHPT model, we should consider λ as a fitting parameter that is just needed to reproduce experimental values of $\phi_{c}$. It is also important to note that the use of protein specific volume to convert concentrations into volume fractions is debatable as the diameter of a protein is not straightforwardly connected to protein specific volume. This adds uncertainty in the comparison between the theoretical and experimental values of $\phi_{c}$. In some studies [33,63], the protein diameter has also been proposed as an extra fitting parameter that is varied to enhance model accuracy.

It is known that the range of interactions specifies the value of $\phi_{c}$, with λ≈1.3 corresponding to $\phi_{c}\approx 0.20$ [24]. Thus, we focus on the LLPS boundary extracted using Equations (8)–(12) with λ=1.3. For comparison, the critical point extracted from related Monte Carlo simulations [24] is also included. We can appreciate that the values of $\beta_{c}^{-1}$ and $\phi_{c}$ calculated using BHTP are both ≈6% higher than those extracted from simulations. For completeness, we also included the boundary calculated after setting $a_{R2}=$ 0. This exhibits relatively larger deviations from simulation results, as expected.

**Figure 4:**
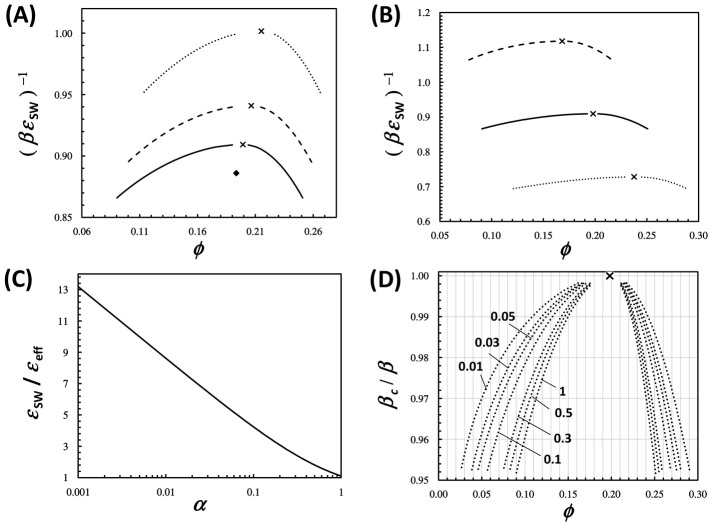
(**A**) Phase diagram showing normalized temperature, ${\left(\beta \varepsilon_{\mathrm{SW}}\right)}^{-1}$, as a function of protein volume fraction, ϕ. LLPS boundaries extracted using λ= 1.3, α= 1, and $a_{R2}$ calculated using Equation (12) (dashed curve), Equation (13) (solid curve), and setting $a_{R2}=$ 0 (dotted curve). Diamond indicates critical point extracted from Monte Carlo simulations. (**B**) Phase diagram showing LLPS boundaries extracted using α= 1 and λ= 1.2 (dotted curve), 1.3 (solid curve), and 1.4 (dashed curve). (**C**) Plot describing dependence of $\varepsilon_{\mathrm{SW}}/\varepsilon_{\mathrm{eff}}$ on α at the critical temperature with λ= 1.3. (**D**) Phase diagram showing how the width of LLPS boundaries depend on α at λ= 1.3. The number associated with each curve is the corresponding values of α.

It is important to note that Equation (12) does not yield the correct expression of the second virial coefficient, B, for the square-well fluid [65]. Specifically, it yields $B/4=1-(\lambda^{3}-1)(\beta \varepsilon_{\mathrm{SW}}+\beta^{2}\varepsilon_{\mathrm{SW}}^{2}/2)]$ instead of $B/4=1-(\lambda^{3}-1)(e^{\beta \varepsilon_{\mathrm{SW}}}-1)]$. Since the second virial coefficient is a fundamental thermodynamic property of fluids, it is important that the expression of $a_{R2}$ reproduces the correct expression of B. We therefore replace Equation (12) with:

(13) $$a_{R2}=-\frac{1}{2}\phi^{2}\frac{d\nu_{\mathrm{HS}}}{d\pi_{\mathrm{HS}}}(e^{\beta \varepsilon_{\mathrm{SW}}}-1-\beta \varepsilon_{\mathrm{SW}})$$

which is the same as Equation (12) to second order in $\beta \varepsilon_{\mathrm{SW}}$. As shown in Figure 4A, Equation (13) significantly improves agreement with the Monte Carlo simulations. Specifically, the values of $\beta_{c}^{-1}$ and $\phi_{c}$ are now found to be just ≈2% higher than those extracted from the simulations. Hence, we use Equation (13) instead of Equation (12) in our data analysis. In Figure 4B, we show the LLPS boundaries calculated at different values of λ. Here, we can see that $\phi_{c}$ increases as λ decreases, as expected from theory and Monte Carlo simulations [24].

We now need to incorporate anisotropy in the BHPT model. One way to approximately describe anisotropy in the square-well fluid is to replace $\varepsilon_{\mathrm{SW}}$ with a temperature-dependent effective energy, $\varepsilon_{\mathrm{eff}}$, given by the following free energy expression [53]:

(14) $$\beta \;\varepsilon_{\mathrm{eff}}/2=\;\ln[\alpha \;e^{\;\beta \;\varepsilon_{\mathrm{SW}}/2}+(1-\alpha )]$$

where the factor “2” considers that a protein–protein interaction involves two proteins, α is a degeneracy factor representing the fraction of particle surface occupied by binding sites with energy $\varepsilon_{\mathrm{SW}}$, and 1−α represents the fraction of particle surface with zero binding energy. If α=1, we recover the isotropic case: $\varepsilon_{\mathrm{eff}}=\varepsilon_{\mathrm{SW}}$. As α decreases, the area covered by binding sites decreases and the anisotropic character of protein–protein interactions increases.

### 2.4. Effect of HEPES on Lysozyme LLPS Boundary

**Figure 5:**
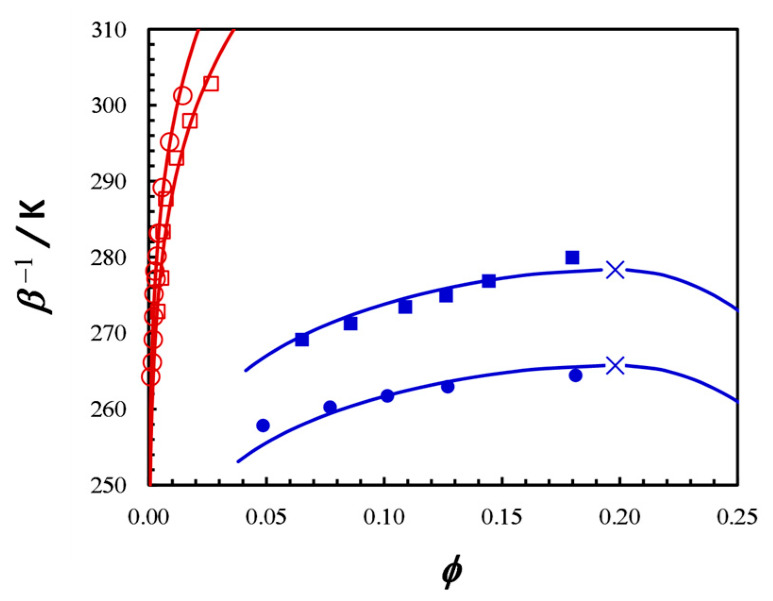
Phase diagram showing experimental LLPS data for the HEPES (solid circles) and REF (solid squares) systems. Theoretical LLPS boundaries are calculated from our BHPT model using λ = 1.3 in both cases. The values of $\varepsilon_{\mathrm{SW}}=$ 1732 K and α=0.0293, and $\varepsilon_{\mathrm{SW}}=$ 1687 K and α=0.0372 are used for the HEPES and REF systems, respectively. Solubility data for the HEPES (open circles) and REF (open squares) systems are also included. Theoretical solubility curves are obtained using the BHPT model for the fluid phase and cell model for the solid phase, with $\Omega_{S}=2.6\times 10^{-6}$, $n_{S}=6$, and $\varepsilon_{S}=\varepsilon_{\mathrm{SW}}$.

We now employ the BHTP model with λ = 1.3 to describe the experimental LLPS boundaries for the REF and HEPES systems. These are shown in the phase diagrams of Figure 5 together with the corresponding crystal solubility curves. To make the crystal model consistent with the BHPT model, we set the attraction energy parameter, $\varepsilon_{\mathrm{SW}}$, for the fluid phase to be the same as $\varepsilon_{S}$ extracted from solubility data, in line with previously reported colloid models of proteins [31,48]. In other words, protein–protein contacts in the crystal lattice are assumed to occur within the square-well range of λ = 1.3. In this way, the thermodynamic description of the fluid phase remains consistent with that of the solid phase.

We are then left with identifying the values of α that best fit the two sets of LLPS data. We determine that α=0.037 describes the LLPS of the REF system well. This value must then be decreased to α=0.027 in order to accurately represent the lower LLPS temperatures of the HEPES system. Notably, these values of α also describe the width of the LLPS boundaries fairly well. Specifically, experimental LLPS data show a decrease of ≈4% in LLPS temperature when the protein volume fraction changes from ≈0.2 to 0.05. These variations are comparable with those shown for α=0.03 in Figure 4D.

Although the accurate interpretation of the obtained values of α is hampered by the simplifications introduced in this colloid model, it remains useful to provide a physical interpretation of this parameter. For example, we may estimate how a directional cross-linking interaction reduces the area of one of the six binding regions discussed in Section 2.2. Since the accessible surface area of lysozyme is $\approx 80\;{\mathrm{nm}}^{2}$ (protein data bank, pdb 1E8L) [66], we use the α values to estimate that the average area of a binding region is $\approx 50\;{Å}^{2}$ and $\approx 40\;{Å}^{2}$ for the REF and HEPES systems, respectively. For comparison, we expect that the area associated with a cross-linking interaction should be $\approx 10\;{Å}^{2}$ (area with a radius on the order of a hydrogen bond). Hence, we may describe the reduction in α by assuming that one binding region reduces its area from $\approx 100\;{Å}^{2}$ to $\approx 10\;{Å}^{2}$ due to HEPES.

In Figure 5, theoretical solubility curves are now obtained by calculating the protein chemical potential using the BHPT model. These theoretical curves, which are also shown in Figure 5, accurately describe solubility data, justifying the initial use of $\hat{\mu }\approx {\hat{\mu }}_{0}+\ln\phi$. Finally, we can also make predictions on the values of the second virial coefficient at 298 K. Specifically, we use Equation (14) to calculate that $\varepsilon_{\mathrm{eff}}=$ 278 K for the REF system is higher than $\varepsilon_{\mathrm{eff}}=$ 244 K for the HEPES system. If we then use the square-well expression of the second virial coefficient with $\varepsilon_{\mathrm{eff}}$ replacing $\varepsilon_{\mathrm{SW}}$:

(15) $$B/4=1-(\lambda^{3}-1)(e^{\;\beta \;\varepsilon_{\mathrm{eff}}}-1)$$

we calculate B= −3.4 for the REF system and the higher value of B= −2.1 for the HEPES system. Their difference, +1.3, is essentially the same as that estimated from DLS data at 298 K (+1.2±0.4).

## 3. Conclusions

We applied a colloid model to theoretically examine the opposite effects of HEPES on lysozyme crystal solubility and the LLPS boundary. We specifically applied cell theory to solubility data to determine that HEPES increases protein–protein attraction energy by 2.7%. To explain the observed decrease in the LLPS temperature, we considered that HEPES also enhances the anisotropic character of protein–protein interactions. We developed an analytical model based on BHPT to describe the LLPS boundaries using the same protein–protein attraction energy parameter extracted from solubility data ($\varepsilon_{\mathrm{SW}}=\varepsilon_{S}$). Only the parameter α was decreased in order to increase anisotropy and successfully explain the effect of HEPES on LLPS temperature and change in the second virial coefficient. Our work describes a useful analytical model for describing the multifaceted effects of additives on the phase diagram of protein solutions.

## 4. Methods

The LLPS boundary is represented by pairs of volume fractions, $\phi^{\left(I\right)}(\beta )$ and $\phi^{\left(\mathrm{II}\right)}(\beta )$ at various values of sufficiently high β. The software MATLAB (version R2010a) was employed to carry out all calculations. Starting from a low β, the chosen expression of $a=a_{\mathrm{HS}}+a_{R}$ (with ${\hat{\mu }}_{0}=0$) is numerically differentiated with respect ϕ to extract $\hat{\mu }(\beta ,\phi )$ from Equation (1a) and then $\hat{\pi }(\beta ,\phi )$ from Equation (1b). If the plot of $\hat{\pi }$ as a function of ϕ is monotonic, β is increased until non-monotonic behavior emerges, and the minimum of $\hat{\pi }$ at $\phi =\phi_{\min}$ is identified. At this β value, Equation (2a,b) are numerically solved to extract $\phi^{\left(I\right)}$ and $\phi^{\left(\mathrm{II}\right)}$. Specifically, a value of ϕ slightly larger than $\phi_{\min}$ is then chosen as the initial seed of $\phi^{\left(\mathrm{II}\right)}$ and the corresponding $\hat{\mu }(\beta ,\phi^{\left(\mathrm{II}\right)})$ is calculated. At this value of $\hat{\mu }$, the corresponding $\phi^{\left(I\right)}$ is computed by applying Newton’s method starting with a very low initial seed of $\phi^{\left(I\right)}$ (e.g., 0.0001). The extracted value of $\phi^{\left(I\right)}$ is then used to calculate $\hat{\pi }(\beta ,\phi^{\left(I\right)})$. At this value of $\hat{\pi }$, the corresponding $\phi^{\left(\mathrm{II}\right)}$ is computed by applying Newton’s method starting with a very high initial seed of $\phi^{\left(\mathrm{II}\right)}$ (0.74). This value of $\phi^{\left(\mathrm{II}\right)}$ is, in turn, used to calculate $\hat{\mu }(\beta ,\phi^{\left(\mathrm{II}\right)})$ again. This iterative procedure is repeated until convergence is achieved and $\phi^{\left(I\right)}(\beta )$ and $\phi^{\left(\mathrm{II}\right)}(\beta )$ are extracted. The value of β is then increased and the same iterative procedure is repeated, producing new pairs of $\phi^{\left(I\right)}(\beta )$ and $\phi^{\left(\mathrm{II}\right)}(\beta )$. Our method is sufficiently fast that computations could be repeated at increasing precision so that computational errors become ultimately negligible. Critical coordinates, $\left(\phi_{c},\beta_{c}\right)$, are extracted by extrapolating β and $(\phi^{\left(I\right)}+\phi^{\left(\mathrm{II}\right)})/2$ to ${\left(\phi^{\left(\mathrm{II}\right)}-\phi^{\left(I\right)}\right)}^{2}\to 0$.

The crystal solubility boundary is described by the protein volume fraction of the coexisting fluid phase, $\phi_{S}(\beta )$. At any given β, ${\hat{\mu }}_{S}$ is calculated using Equation (6), while $\hat{\mu }(\beta ,\phi )$ for the fluid phase is obtained by differentiation of a using Equation (1a). We can then extract $\phi_{S}$ by numerically solving Equation (5). Specifically, we apply Newton’s method starting with a very low initial seed of ϕ, until convergence is achieved yielding $\phi_{S}$.

## Data Availability

The original contributions presented in this study are included in the article. Further inquiries can be directed to the corresponding author.

## Supplementary Materials

The following supporting information can be downloaded at: https://www.mdpi.com/article/10.3390/molecules31111894/s1, Section S1: Wertheim Perturbation Theory (WPT); Section S2: Effect of HEPES on lysozyme LLPS boundary from WPT; Section S3: Second Virial Coefficient from WPT.
