# Supplementary material for: Modeling Opposite Effects of an Additive on Liquid–Liquid Phase Separation and Crystal Solubility of Protein Solutions
Source: Molecules. 2026 Jun 1;31(11):1894. doi: 10.3390/molecules31111894 (PMC13258223; doi:10.3390/molecules31111894)
Supplement: Supplementary file 1 [file molecules-31-01894-s001.zip › molecules-4318026-supplementary.pdf]

Modeling Opposite Effects of an Additive on Liquid-Liquid Phase Separation and Crystal Solubility of Proteins

Onofrio Annunziata and Shamberia Thomas

Department of Chemistry and Biochemistry, Texas Christian University, Fort Worth, TX 76109, USA

Section S1. Wertheim Perturbation Theory (WPT)

To describe LLPS, we also examined Wertheim perturbation theory of associating spheres (WPT) The residual Helmholtz free energy for this model is [33,48,60]:

$$a_{\mathrm{R}}(\beta,\phi) = n_b\,\phi\left(\ln X + \frac{1-X}{2}\right) \tag{S1}$$

where n_b is the number of binding sites, which is assumed identical and independent. These sites are randomly distributed on particle surface. Binding between two particles is modeled using a square-well potential with attraction energy, ε_b. Binding occurs only when the distance between the surface sites of two different particles is less than the interaction range, δ (as a multiple of particle diameter, σ). To ensure that binding does not involve more than two proteins, we must have: $\delta < 1-\sqrt{3}/2 \cong 0.134$. In Eq. S1, X is the fraction of free sites, with $a_{\mathrm{R}} = 0$ when $X = 1$. It is obtained from the mass-action equation [48]:

$$X = \frac{1}{1+\omega X} \tag{S2}$$

or

$$X = \frac{2}{1+\sqrt{1+4\omega}} \tag{S3}$$

where

$$\omega \equiv \frac{n_b\phi}{V_{\mathrm{P}}}\int_{\sigma}^{(1+\delta)\sigma}(4\pi r^2)\cdot f\cdot g_{\mathrm{HS}}\,dr = 24 n_b\phi\int_{1}^{1+\delta} x^2\cdot f\cdot g_{\mathrm{HS}}\,d\,x \tag{S4}$$

with $x \equiv r/\sigma$, $V_{\mathrm{P}} = (\pi/6)\sigma^3$ and

$$f = (e^{\beta\varepsilon_b}-1)\frac{1}{6\sigma^2 r}(\sigma\delta+\sigma-r)^2(2\sigma\delta-\sigma+r) = (e^{\beta\varepsilon_b}-1)\frac{(1+\delta-x)^2}{6}\left(1-\frac{1-2\delta}{x}\right) \tag{S5}$$

The function, $f(x)$, is an angle average of the site-site Mayer function [33,63]. In Eq. S4, $g_{\mathrm{HS}}(x,\phi)$ is the pair distribution function of the hard-sphere reference fluid. If we insert Eq. S5 into Eq. S4,

we obtain

$$\omega = 4n_b\phi\,(e^{\beta\varepsilon_b}-1)\int_1^{1+\delta}(1+\delta-x)^2\left(1+\frac{2\delta-1}{x}\right)g_{\mathrm{HS}}(x,\phi)x^2dx \tag{S6}$$

We can formally rewrite Eq. S6 in the following way:

$$\omega = n_b\phi\,(e^{\beta\varepsilon_b}-1)g_{\mathrm{HS}}(1,\phi)\,\Omega_b \tag{S7}$$

where

$$g_{\mathrm{HS}}(1,\phi)=\frac{1-\phi/2}{(1-\phi)^3} \tag{S8}$$

is the Carnahan-Starling contact value of the pair distribution function and Ω_b is a phase volume (as a multiple of V_{P}) over which a bond can form. After introducing, the difference, $\Delta g_{\mathrm{HS}}\equiv g_{\mathrm{HS}}(x,\phi)-g_{\mathrm{HS}}(1,\phi)$, it follows from Eq. S6 that the expression of Ω_b is

$$\Omega_b=4\int_1^{1+\delta}(1+\delta-x)^2\left(1+\frac{2\delta-1}{x}\right)x^2dx+4\int_1^{1+\delta}\frac{\Delta g_{\mathrm{HS}}(x,\phi)}{g_{\mathrm{HS}}(1,\phi)}(1+\delta-x)^2\left(1+\frac{2\delta-1}{x}\right)x^2dx \tag{S9}$$

In general, we need an expression for $g_{\mathrm{HS}}(x,\phi)$. For sufficiently short distances, we can write [59]:

$$g_{\mathrm{HS}}(x,\phi)=g_{\mathrm{HS}}(1,\phi)+g'_{\mathrm{HS}}(\phi)\cdot(x-1) \tag{S10}$$

where

$$g'_{\mathrm{HS}}\equiv\left(\frac{\partial g_{\mathrm{HS}}}{\partial x}\right)_\phi\Bigg|_{x=1}=-\frac{9}{2}\frac{1+\phi}{(1-\phi)^3}\phi \tag{S11}$$

Thus, the expression of Ω_b becomes

$$\begin{aligned}\Omega_b&=4\int_1^{1+\delta}(1+\delta-x)^2\left(1+\frac{2\delta-1}{x}\right)x^2dx+\\&-18\frac{1+\phi}{1-\phi/2}\phi\int_1^{1+\delta}(1+\delta-x)^2\left(1+\frac{2\delta-1}{x}\right)(x-1)x^2dx\end{aligned} \tag{S12}$$

After solving the two integrals in Eq. S12, we obtain:

$$\Omega_b = \left(3 + \frac{4}{5}\delta\right)\delta^4 - \frac{9}{2}\frac{1+\phi}{1-\phi/2}\phi\left(\frac{4}{5} + \frac{1}{3}\delta\right)\delta^5 \quad \text{(S13)}$$

The second term in Eq. S13 is may be neglected under the assumption that δ is sufficiently short that the Sticky-limit approximation is good [33,48]. Hence, we can introduce the sticky-limit approximation of Ω_b:

$$\Omega_b = \left(3 + \frac{4}{5}\delta\right)\delta^4 \quad \text{(S14)}$$

To assess accuracy of WPT model, we consider preciously reported Monte Carlo simulation data on critical coordinates, available for $n_b = 5$ and $\delta = 0.119$ [59]. We specifically calculated $a_{\rm R}(\beta,\phi)$ using Eq. S1 with Ω_b given by Eq. S14 (sticky limit) and Eq. S13 (general case), and apply Eq. 2a,b in the manuscript to determine $\phi^{(\rm I)}(\beta)$, $\phi^{(\rm II)}(\beta)$ and $(\phi_{\rm c}, \beta_{\rm c}^{-1})$. For completeness, we also calculate $a_{\rm R}$ using the Percus-Yevick expression of the contact value, $g_{\rm HS}(1,\phi) = (1+\phi/2)/(1-\phi)^2$, in Eq. S7, which was previously [33,63] employed, with Ω_b given by Eq. S14. The comparison between the three calculated phase boundaries is shown in Fig. S1A. Here, we can see that the three different choices of $g_{\rm HS}$ produce curves that are fairly close to each other (deviations of ≈1% in the values of $\beta_{\rm c}^{-1}$). We can also appreciate that the Sticky-limit approximation is sufficiently accurate for $\delta \approx 0.1$ (and less). For convenience, we will use the Carnahan-Starling expression of $g_{\rm HS}(1,\phi)$ as it is consistent with the chosen expression of $a_{\rm HS}$. It is also convenient to introduce the phase volume, Ω_b, as a fixed parameter independent of ϕ. Thus, the sticky-limit approximation for Ω_b given by Eq. S14 will be used.

As we can also see in Fig. S1A, the value of $\beta_{\rm c}^{-1}$ from simulations is just ≈2% higher than that calculated using Eq. 8, showing that WPT accurately describes LLPS temperature. However, the critical volume fraction from simulations, $\phi_{\rm c} = 0.185$ [59], is significantly larger than the WPT value of $\phi_{\rm c} = 0.13$. Since the value of $\phi_{\rm c}$ increases with number of sites, we examined the effect of n_b on $\phi_{\rm c}$ using the WPT model. As shown in Fig. S1B, we can achieve $\phi_{\rm c} \approx 0.18$ only if we increase n_b from 5 to 7. Thus, our analysis indicates that the value of n_b from WPT model is about two units larger than the actual number of sites. Since critical volume fractions of protein solutions

are typically about $\phi_c \approx 0.2$ [24], it follows from Fig. 4B that the best WPT value to describe these mixtures is $n_b \approx 8$. It follows from simulation results that this WPT value of n_b is about two units larger than the actual number of sites, $n_b \approx 6$.

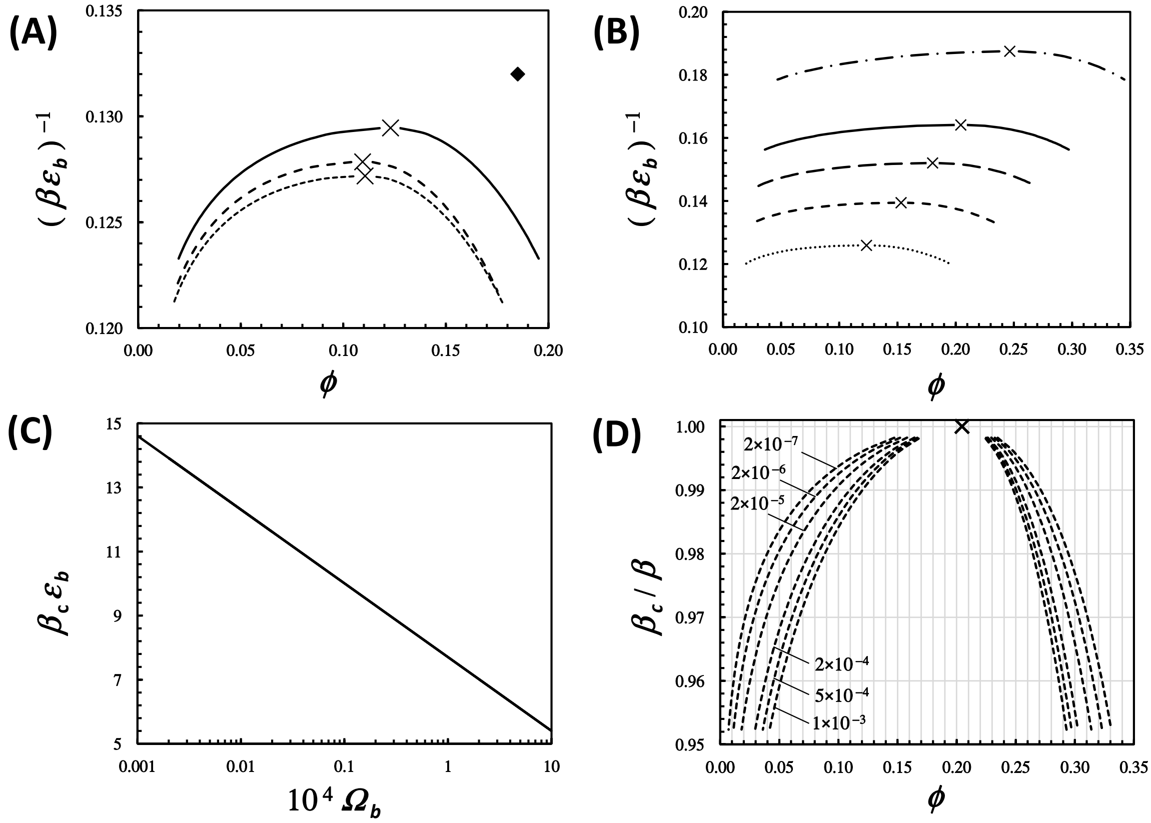

Figure S1. (A) Phase diagram showing normalized temperature, $(\beta\varepsilon_b)^{-1}$, as a function of protein volume fraction, ϕ. LLPS boundaries extracted using $n_b = 5$, $\Omega_b = 6.3\times10^4$ ($\delta = 0.119$) and Carnahan-Starling (solid curve) and Percus-Yevick (dashed curve) values of $g_{\mathrm{HS}}^{\mathrm{con}}$, and integration of $g_{\mathrm{HS}}(r)$ (dotted curve). Crosses indicate the corresponding critical points. Diamond indicates critical point extracted from Monte Carlo simulations. **(B)** Phase diagram showing LLPS boundaries extracted using $n_b = 5$ (dotted curve), 6 (dashed curve), 7 (long dashed curve), 8 (solid curve) and 10 (dash-dotted curve), and $\Omega_b = 5.0\times10^4$. **(C)** Plot describing dependence of $\beta_c\varepsilon_b$ on Ω_b. **(D)** Phase diagram showing how the width of LLPS boundaries depend on Ω_b at $n_b = 8$. Numbers associated with curves are the corresponding values of Ω_b.

In our use of WPT, we should consider n_b as a fitting parameter that is just needed to reproduce experimental values of ϕ_c. It plays the same role as the range of interactions, λ, in the BHPT model. In contrast, the phase volume, Ω_b, does not affect ϕ_c. However, it does have an impact on both critical temperature, β_c^{-1}, and width of LLPS boundary. This is shown in Figs. S1C,D. It follows from Fig. S1C that decreasing Ω_b lowers LLPS temperature (e.g., β_c^{-1}) at constant attraction energy, ε_b. In Fig. S1D, we can appreciate that boundary width increases as Ω_b decreases. These are the characteristic trends associated with an increase in the anisotropic character of protein-protein interactions [24,33,48,53]. Indeed, the orientational degrees of freedom associated with protein-protein binding decrease as the phase volume of a site is reduced. The quantity, Ω_b in the WPT model plays the same role as α in the revised BHPT model.

Section S2. Effect of HEPES on lysozyme LLPS boundary from WPT

We shall now employ the WTP model to describe the experimental LLPS boundaries for REF and HEPES systems. These are shown in the phase diagrams of Fig. S2 together with the corresponding crystal solubility curves. We shall set $n_b = 8$ to accurately reproduce the critical volume fraction of $\phi_c \approx 0.20$. These parameters are assumed to be constant, independent of solvent composition. To make crystal cell model consistent with WPT model, we shall also assume that the attraction energy parameters, ε_b, for the fluid phase are the same as ε_S extracted from solubility data. We are then left with identifying the values of Ω_b that best fit experimental LLPS data. We determine that $\Omega_b = 5.2\times10^{-4}$ describes LLPS of the REF system. This value must be decreased to $\Omega_b = 3.2\times10^{-4}$ in order to accurately represent the lower LLPS temperatures of the HEPES system. Noteworthily, these values of Ω_b also describe the width of the LLPS boundaries fairly well. Finally, theoretical solubility curves are now obtained by calculating the protein chemical potential using WPT and BHPT models. These theoretical curves, which are also shown in Fig. S2, accurately describe solubility data justifying the initial use of $\mu \approx \mu_0 + \ln\phi$.

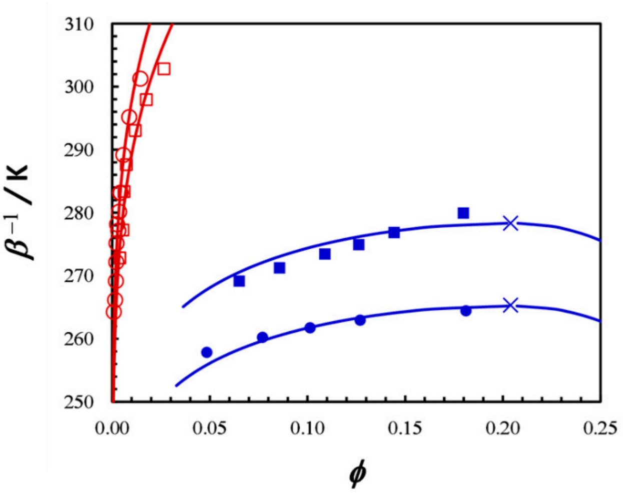

Figure S2. Phase diagram showing experimental LLPS data for the HEPES (solid circles) and REF (solid squares) systems. Theoretical LLPS boundaries are calculated from WPT model with $n_b = 8$ in both cases. The values of $\varepsilon_b = 1732$ K and $\Omega_b = 3.23\times10^{-4}$, and $\varepsilon_b = 1687$ K and $\Omega_b = 5.17\times10^{-4}$ are used for the HEPES and REF systems, respectively. Solubility data for the HEPES (open circles) and REF (open squares) systems are also included. Theoretical solubility curves are obtained using $\Omega_S = 2.6\times10^{-6}$, and $n_S\varepsilon_S = 10390$ K and 10120 K for the HEPES and REF systems, respectively.

Section S3. Second Virial Coefficient from WPT

The second-virial coefficient may be introduced as

$$B = \lim_{\phi\to 0}\frac{\hat{\pi}/\phi - 1}{\phi} = 4 + \lim_{\phi\to 0}\left(\frac{\partial a_{\mathrm{R}}/\phi}{\partial\phi}\right)_{\beta} \quad \text{(S15)}$$

where we have used $\hat{\pi} = \phi[\partial(a/\phi)/\partial\phi]_{\beta}$ from $\Pi = -(\partial A/\partial V)_{\beta}$ and “4” is the second-virial coefficient associated with hard spheres. It follows from Eqs. S1,S2 that:

$$\frac{a_{\mathrm{R}}}{\phi} = n_b\left(\ln\frac{2}{1+\sqrt{1+4\omega}} + \frac{1}{2}\frac{\sqrt{1+4\omega}-1}{1+\sqrt{1+4\omega}}\right) \quad \text{(S16)}$$

where ω is given by Eq. S7 and Ω_b is a constant as described by Eq. S14. In the limit of $\phi\to 0$, we can rewrite Eq. S16 to first order with respect to ω:

$$\frac{a_{\mathrm{R}}}{\phi}=n_b\left(\ln\frac{1}{1+\omega+\ldots}+\frac{1}{2}\frac{\omega+\ldots}{1+\omega+\ldots}\right)=-\frac{n_b}{2}\omega+\ldots \tag{S17}$$

It then follows from Eq. S7 that

$$\lim_{\phi\to 0}\left(\frac{\partial a_{\mathrm{R}}/\phi}{\partial\phi}\right)_\beta=\lim_{\phi\to 0}\frac{1}{\phi}\left(\frac{\partial a_{\mathrm{R}}}{\partial\phi}\right)_\beta=-\frac{n_b^{\ 2}}{2}\cdot\Omega_b\cdot(e^{\beta\varepsilon_b}-1) \tag{S18}$$

where we have also used $g_{\mathrm{HS}}(1,0)=1$. Thus, Eq. S15 becomes:

$$B=4-2n_b^{\ 2}\cdot\Omega_b\cdot(e^{\beta\varepsilon_b}-1) \tag{S19}$$

Finally, we can make predictions on the values of second virial coefficient at 298 K using $n_b=8$ in Eq. S19. Specifically, we use ε_b= 1687 K and $\Omega_b=5.17\times10^{-4}$ to determine that $B=-15.0$ for the REF system, and ε_b= 1732 K and $\Omega_b=3.23\times10^{-4}$ to determine that $B=-9.8$ for the HEPES system at 298 K. These values of B are significantly more negative compared to those obtained from BHPT model. Moreover, their difference at 298 K is +5.2. This is significantly larger than that evaluated from DLS data ($+1.2\pm0.4$) and the revised BHPT model ($+1.3$).

In conclusion, the WPT model can be successfully used to describe the LLPS boundaries using the same protein-protein attraction energy parameter extracted from solubility data (ε_b=ε_S). Only the anisotropy parameter (Ω_b) is decreased in order to successfully explain the effect of HEPES on LLPS temperature. However, the magnitude of the calculated second virial coefficients is relatively large.
